# Effect of β_2_-adrenergic receptor gene (*ADRB2)* 3′ untranslated region polymorphisms on inhaled corticosteroid/long-acting β_2_-adrenergic agonist response

**DOI:** 10.1186/1465-9921-13-37

**Published:** 2012-05-04

**Authors:** Helen J Ambrose, Rachael M Lawrance, Carl J Cresswell, Mitchell Goldman, Deborah A Meyers, Eugene R Bleecker

**Affiliations:** 1 AstraZeneca, Alderley Park, UK; 2 AstraZeneca LP, Wilmington, DE, USA; 3 Wake Forest University Health Sciences, Medical Center Blvd, Winston-Salem, NC, 27157, USA

**Keywords:** Asthma, β_2_-agonist, Inhaled corticosteroid, Genotype, Polymorphism, β_2_-adrenergic receptor, 3′ untranslated region, Poly-C repeat

## Abstract

**Background:**

Evidence suggests that variation in the length of the poly-C repeat in the 3′ untranslated region (3′UTR) of the β_2_-adrenergic receptor gene (*ADRB2*) may contribute to interindividual variation in β-agonist response. However, methodology in previous studies limited the assessment of the effect of sequence variation in the context of poly-C repeat length. The objectives of this study were to design a novel genotyping method to fully characterize sequence variation in the *ADRB2* 3′UTR poly-C repeat in asthma patients treated with inhaled corticosteroid and long-acting β_2_-adrenergic agonist (ICS/LABA) combination therapy, and to analyze the effect of the poly-C repeat polymorphism on clinical response.

**Methods:**

In 2,250 asthma patients randomized to treatment with budesonide/formoterol or fluticasone/salmeterol in a six-month study (AstraZeneca study code: SD-039-0735), sequence diversity in the *ADRB2* poly-C repeat region was determined using a novel sequencing-based genotyping method. The relationship between the poly-C repeat polymorphism and the incidence of severe asthma exacerbations, and changes in pulmonary function and asthma symptoms from baseline to the average during the treatment period, were analyzed.

**Results:**

Poly-C repeat genotypes were assigned in 97% (2,192/2,250) of patients. Of the 13 different poly-C repeat alleles identified, six alleles occurred at a frequency of >5% in one or more population in this study. The repeat length of these six common alleles ranged from 10 to 14 nucleotides. Twelve poly-C repeat genotypes were observed at a frequency of >1%. No evidence of an association between poly-C repeat genotype and the incidence of severe asthma exacerbations was observed. Patients’ pulmonary function measurements improved and asthma symptoms declined when treated with ICS/LABA combination therapy regardless of poly-C repeat genotype.

**Conclusions:**

The extensive sequence diversity present in the poly-C repeat region of the *ADRB2* 3′UTR did not predict therapeutic response to ICS/LABA therapy.

## Background

Results from studies in patients with asthma have shown considerable variation in the therapeutic response to β_2_-adrenergic agonists [1-3], which may be associated with genetic variation in the β_2_-adrenergic receptor gene (*ADRB2*) [4-7]. In some studies, a difference in clinical response to short-acting β_2_-adrenergic agonist (SABA) and long-acting β_2_-adrenergic agonist (LABA) therapies has been observed in asthma patients with specific *ADRB2* genotypes [8-11]. Recent pharmacogenetic analyses have shown no effect of *ADRB2* variation on clinical response to inhaled corticosteroid (ICS)/LABA combination therapy [12-15]. However, these studies did not explore the effect of diversity in the poly-C repeat on the 3′ untranslated region (3′UTR) of *ADRB2*.

It has been proposed that genetic polymorphisms in the 3′UTR of *ADRB2* may contribute to variation in response to β_2_-adrenergic agonist therapy [16]. A repeat length polymorphism has been identified in a ‘C’ nucleotide-rich region 23 bp downstream from the translation stop codon of *ADRB2* in the poly-C repeat region [16]. Results from a case-control study in a multiethnic population showed a significant association between the single-nucleotide polymorphism (SNP) rs1042714 (encoding the Gln27Glu variation) and an *ADRB2* haplotype extending across the poly-C repeat region, with the asthma phenotype in African Americans [16]. This study suggested that the length of the poly-C repeat may affect pulmonary function and thus contribute to interindividual variation in β-agonist response. Findings from another study showed that patients with the Arg16Arg polymorphism who have tracts of 11C’s in the poly-C region have reduced *ADRB2* expression and increased agonist-promoted downregulation compared with those with other poly-C lengths [17]. The authors proposed that these factors may result in decreased bronchodilator response and tachyphylaxis to inhaled β_2_-agonists [17].

The fragment-size−based genotyping method used by Hawkins and coworkers to characterize the 3′UTR poly-C repeat polymorphism [16] does not allow substitutions that interrupt the poly-C repeat to be characterized in the context of poly-C repeat length. Therefore, the objective of the present study was to design a novel and robust genotyping method to fully characterize sequence variation in the *ADRB2* 3′UTR poly-C repeat region in 2,250 patients previously treated with ICS/LABA therapy, and to analyze the effect of the poly-C repeat polymorphism on clinical response to ICS/LABA combination therapy. It was hypothesized that sequence variation in the 3′UTR region of the *ADRB2* gene may affect response to therapy and the incidence of exacerbations. In this report, the poly-C allele is defined as all variation in the poly-C repeat region (+1266 to +1278), including length of poly-C repeat and any additional substitutions. The poly-C genotype is the combination of two alleles in any one individual.

## Methods

### Study population and clinical measurements

Patients included in the present study were participants in a previously described clinical study (SD-039-0735) [18] and pharmacogenetic analysis [13]. Briefly, the study population included 2,250 patients with asthma who were randomized to either budesonide/formoterol dry powder inhaler (DPI) 160/4.5 μg × 1 inhalation twice daily, budesonide/formoterol DPI 320/9 μg × 1 inhalation twice daily, or fluticasone/salmeterol DPI 125/25 μg × 2 inhalations (250/50 μg) twice daily for six months [13,18]. The study protocol was approved by independent ethics committees for the 235 sites within 16 countries participating in the study, and all patients included in these analyses provided informed consent for genetic analysis. Outcomes analyzed in this study included the number of severe asthma exacerbations (defined as deterioration in asthma leading to hospitalization, emergency department treatment, or oral glucocorticosteroid treatment lasting three or more days) and changes in pulmonary function measurements (forced expiratory volume in one second [FEV_1_ and morning peak expiratory flow [PEF], total symptom scores, use of rescue medication, and nighttime awakenings) from baseline to the average during the treatment period. Race was self-reported; 73% of patients were white of European descent (n = 1,614), 7% were Asian (n = 156), 1% were black (n = 21), and 19% were of mixed or other race (n = 434).

### DNA sequencing

**Figure 1:**
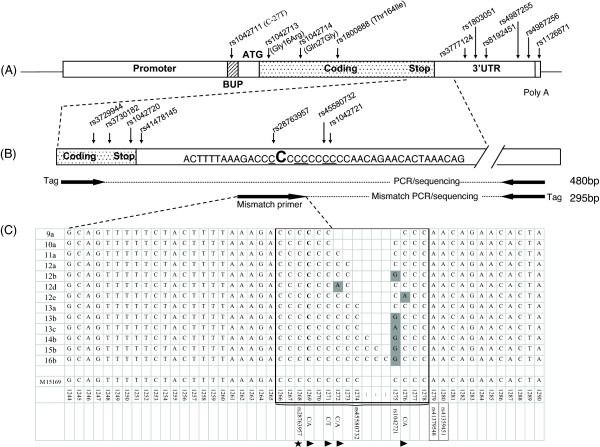
***ADRB2*3΄UTR poly-C repeat region.** (**A**) Structural overview of the *ADRB2* gene, detailing the common amino acid changing SNPs, and flanking variants (↓). Annotated are the *ADRB2* upstream peptide (BUP), ATG start codon, coding region (shaded), 3′ untranslated region (3'UTR), and polyA addition signal. (**B**) Expanded view of the *ADRB2* 3′UTR immediately downstream of the stop codon. SNPs located at the end of the coding sequence, as well as those located within the poly-C repeat region, are shown (↓). 3′UTR SNPs identified in this study are underlined. The C nucleotide that is mismatched in the sequencing primer to a T nucleotide (see Methods) is highlighted in larger bold font (position +1269). Below the sequence, arrows indicate the position of the primers described in the text (see Methods). Sizes of the PCR products, including sequencing tags, are shown at the right-hand side. (**C**) An expanded view showing detail of the variable nature of the *ADRB2* 3'UTR. The poly-C repeat region (rs45580732) is boxed with internal SNPs highlighted by shaded nucleotide bases. The location of the mismatch primer is indicated by the dotted lines expanding out the arrow in Figure 1b. ★The C > G SNP at position +1268 was previously described by Hawkins et al. (2006) [16]. ► indicates novel variants identified in this study. SNP rs IDs enclosed in dashed boxes below the table are database variants that were not seen among the chromosomes sequenced in this study.

A novel sequence-based genotyping approach was used in this study to investigate and genotype sequence diversity in the poly-C region of *ADRB2* because the 3′UTR was refractory to standard polymerase chain reaction (PCR) sequencing. This method interrupted poly-C repeats and disrupted secondary structure with mismatched PCR primer in which the ‘C’ nucleotide was changed to ‘T’ (Figure 1). Additional details are provided in the Additional file 1.

### Statistical methods

As in previous analyses [13], patients of various races on various ICS/LABA combination treatments were combined in the present exploratory analysis to assess the association of the large number of poly-C genotypes with ICS/LABA treatment outcomes in a large patient population. Demographics and baseline patient characteristics during run-in were summarized by poly-C repeat genotype group for all groups present at a frequency of >1%. The incidence of severe asthma exacerbations was summarized by poly-C genotype group. The effect of poly-C repeat genotype on time to first exacerbation was analyzed using a Cox proportional hazards model, and the proportion of patients with exacerbations in each poly-C genotype group was compared using an ordinal logistic regression model. Changes in FEV_1_, morning PEF, total daily asthma symptom scores, total daily rescue medication use, and nighttime awakenings, from run-in to the average over the treatment period, were compared using analysis of variance (ANOVA) models. ANOVA models included poly-C genotypes (frequency >1%), treatment, country, and baseline value. In a previous pharmacogenetic analysis, there was no effect of the *ADRB2* Gly16Arg polymorphism on pulmonary function [13]; however, because of interest in the effect of the Gly16Arg SNP, changes from baseline in FEV_1_ and morning PEF were assessed using additional ANOVA models, including Gly16Arg genotype as a covariate in whites of European descent and Asian populations.

## Results

Allelic variation in the poly-C repeat region (from positions +1266 to +1278 bp relative to the ATG start codon in *ADRB2* reference sequence M15169.1) was determined through sequencing of PCR products generated using a mismatched PCR primer. The mismatched primer, which hybridizes to sequences between position +1244 and +1271 (Figure 1), introduced a ‘T’ nucleotide at position +1269. To ensure polymorphisms underlying the mismatched PCR primer did not affect poly-C genotype determination, the region was re-sequenced in all patients using a PCR product generated using an upstream forward primer (see online repository). A rare polymorphism has been previously identified within the mismatched primer binding site at +1268 (rs28763957) [19]. In the patients in this study, this SNP was found to be triallelic; whites of European descent C1268C, n = 1617; C1268G, n = 4; C1268A, n = 5; and undetermined, n = 8 (allele frequency in whites of European descent 1268G = 0.12%, 1268A = 0.15%). In addition, 11 patients of mixed race were heterozygous C1268G. A novel SNP at C1269A was identified in one mixed-race patient. A novel C1271T polymorphism was identified; one black patient and five patients of mixed race were found to be heterozygous C1271T, and one black patient was a rare homozygote T1271T. One mixed-race patient was heterozygous for both the C1268G and C1271T polymorphisms.

In total, 27 patients (1.2%) were identified with polymorphisms within the mismatched primer region and thus were excluded from the determination of the poly-C genotype. In addition, poly-C genotype was undetermined in a further 31 (1.4%) patients due to failure to sequence the mismatched PCR product. Therefore, 97% of patients (2192 of 2250) were assigned poly-C genotypes.

Extensive diversity in the poly-C repeat region was found; 13 poly-C alleles were identified (Figure 1). Clear differences in the poly-C allele frequencies were observed between the whites of European descent and Asian and black populations in this study. Six of the poly-C alleles were present at a frequency of >5% in one or more population groups (alleles 10a, 11a, 12a, 13b, 13c, and 14b) (Table 1). These common poly-C alleles included repeats of the ‘C’ nucleotide between 10 and 14 bases. Poly-C alleles 13b and 14b have a ‘G’ nucleotide within the poly-C repeat region at position +1275 (rs1042721). Poly-C allele 11a was the most common allele in whites of European descent (36%) but was relatively rare in Asians (2%). In the Asian population, poly-C allele 13b was the most frequent (41%). Poly-C allele 12a was relatively common in the whites of European descent (24%) and Asian (31%) populations. The frequency of alleles 10a and 14b was greater in the Asian population (13% and 11%, respectively) than in whites of European descent (4% and 6%, respectively). There were 12 poly-C genotypes that were observed at a frequency of >1% in this study; 95% of whites of European descent and 88% of Asian patients had one of these genotypes (Table 2).

**Table 1:** Allele frequencies

	**White**^**a**^	**Asian**	**Black**	**Mixed**	**Total**
Patients, n	1,634	156	21	439	2,250
No. of alleles determined Alleles determined, %	3,216	310	38	820	4,384
9a	0.1	–	–	0.1	0.1
10a	3.6	13.2	21.1	3.5	4.4
11a	35.6	2.3	13.2	19.5	30.0
12a	23.5	31.3	7.9	28.9	24.9
12b	<0.1	–	–	–	<0.1
12d	–	–	–	0.1	<0.1
12e	–	0.3	–	–	<0.1
13a	0.9	1.0	2.6	0.6	0.8
13b	30.0	40.6	39.5	37.3	32.2
13c	<0.1	–	5.3	1.1	0.3
14b	6.0	10.6	10.5	8.5	6.8
15b	0.1	0.6	–	0.2	0.1
16b	0.2	–	–	–	0.1
No. of alleles undetermined	52	2	4	58	116

^a^Whites of European descent.

Allele frequency calculated from the number of alleles determined. Where the allele was not observed at all, ‘-’ was used.

**Table 2:** Patient demographics at baseline and characteristics during the study run-in period by poly-C repeat genotypes

**Poly-C genotype**	10a/11a	10a/12a	11a/11a	10a/13b	11a/12a	11a/13b	12a/12a	11a/14b	12a/13b	12a/14b	13b/13b	13b/14b	Genotypes <1%	Undetermined
Patients, n (%)	44 (2)	57 (3)	225 (10)	62 (3)	314 (14)	418 (19)	136 (6)	74 (3)	357 (16)	77 (3)	211 (9)	119 (5)	98 (4)	58 (3)
Ethnic origin, n (%)														
White^a^	39 (2)	33 (2)	204 (12)	32 (2)	277 (17)	349 (21)	86 (5)	59 (4)	212 (13)	53 (3)	141 (9)	72 (4)	51 (3)	26 (2)
Black	2 (10)	0	1 (5)	4 (19)	0	1 (5)	0	0	2 (10)	0	2 (10)	2 (10)	5 (24)	2 (10)
Asian	0	12 (8)	0	15 (10)	4 (3)	3 (2)	11 (7)	0	48 (31)	8 (5)	21 (13)	15 (10)	18 (12)	1 (1)
Other	3 (1)	12 (3)	20 (5)	11 (3)	33 (8)	65 (15)	39 (9)	15 (3)	95 (22)	16 (4)	47 (11)	30 (7)	24 (5)	29 (7)
Men, n (%)	17 (1)	25 (1)	98 (4)	28 (1)	134 (6)	183 (8)	52 (2)	37 (2)	132 (6)	29 (1)	78 (3)	45 (2)	53 (2)	20 (1)
Age, mean (SD), years	39.9 (15.4)	36.1 (15.8)	39.9 (15.9)	34.8 (16.5)	39.5 (16.7)	39.1 (17.4)	36.5 (16.7)	38.6 (17.0)	37.3 (17.1)	35.2 (16.8)	35.9 (15.8)	39.0 (16.8)	34.5 (16.3)	35.6 (17.1)
FEV_1_, mean (SD), L	2.2 (0.6)	2.2 (0.6)	2.3 (0.6)	2.2 (0.7)	2.3 (0.7)	2.3 (0.7)	2.2 (0.6)	2.3 (0.7)	2.2 (0.7)	2.3 (0.6)	2.2 (0.7)	2.1 (0.8)	2.2 (0.7)	2.1 (0.6)
FEV_1_, mean (SD), % predicted	73.6 (14.3)	72.3 (12.3)	73.4 (13.4)	70.9 (13.4)	73.7 (14.1)	73.1 (12.9)	72.7 (12.6)	73.5 (11.5)	72.5 (13.5)	74.2 (13.1)	72.3 (13.9)	73.1 (16.0)	69.5 (12.8)	70.7 (13.5)
FEV_1_ reversibility, mean (SD), %	25.6 (16.4)	24.2 (11.6)	23.6 (12.9)	22.2 (11.0)	24.4 (12.3)	24.0 (12.2)	25.4 (12.6)	24.5 (12.9)	25.6 (14.3)	26.4 (16.6)	24.7 (11.7)	22.9 (10.8)	24.1 (13.9)	26.5 (12.8)
FVC, mean (SD), L	2.9 (0.7)	3.0 (0.8)	3.2 (1.0)	2.9 (0.9)	3.2 (1.0)	3.1 (0.9)	3.0 (0.9)	3.1 (0.9)	2.9 (1.0)	3.0 (0.9)	3.0 (1.0)	2.9 (0.9)	3.0 (1.0)	2.8 (0.7)
ICS dose at study entry, mean (SD), μg per day	747.7 (261.9)	750.0 (195.5)	766.0 (249.6)	796.0 (232.6)	734.1 (216.4)	749.0 (243.6)	701.8 (207.0)	776.4 (239.0)	708.0 (221.5)	734.4 (254.7)	745.3 (250.5)	694.5 (189.3)	713.8 (191.9)	736.2 (236.0)
Morning PEF during run-in, mean (SD), L/min	322.1 (88.7)	331.4 (103.5)	341.6 (98.1)	338.4 (99.6)	340.7 (92.7)	334.9 (99.0)	337.8 (82.3)	350.5 (95.1)	321.6 (92.0)	342.2 (90.6)	333.1 (85.6)	320.3 (98.3)	358.7 (106.8)	316.5 (76.7)
Total rescue medication use during run-in, mean (SD), inhalations/day	2.2 (1.1)	2.5 (1.6)	2.5 (1.6)	2.5 (1.4)	2.3 (1.4)	2.4 (1.5)	2.2 (1.5)	2.4 (1.4)	2.2 (1.2)	2.1 (1.1)	2.3 (1.2)	2.4 (1.3)	2.2 (1.3)	3.1 (2.2)
Nights with awakenings during run-in, mean (SD), %	32.6 (35.9)	33.8 (33.8)	31.8 (34.6)	33.5 (33.7)	35.7 (35.0)	32.2 (34.0)	33.5 (35.5)	34.5 (37.0)	33.4 (35.1)	35.7 (36.9)	33.7 (35.8)	29.8 (35.1)	32.9 (36.7)	47.1 (35.6)
Total symptom score, mean (SD)	1.9 (0.7)	2.0 (0.9)	1.9 (0.9)	1.9 (0.9)	2.0 (0.9)	2.0 (0.9)	1.9 (1.0)	2.0 (0.9)	1.9 (0.9)	1.8 (0.7)	2.0 (0.9)	2.1 (1.0)	1.9 (1.0)	2.2 (1.1)

^a^Whites of European descent.

Baseline and run-in characteristics for patients of all races. Genotypes present at a frequency of <1% are grouped together, and patients whose poly-C genotype was undetermined are grouped together. *FEV*_*1*_ = forced expiratory volume in one second; *FVC* = forced vital capacity; *ICS* = inhaled corticosteroid; *PEF* = peak expiratory flow; *SD* = standard deviation.

In addition to the substitutions note in the poly-C repeat region, an additional novel low-frequency indel was found immediately 3΄ of the poly-C tract at position +1280 (Figure 1). An insertion of one ‘A’ nucleotide was found on the background of allele 10a (seen four times: one white of European descent, one black, and two mixed-race individuals), and the insertion of two ‘A’ nucleotides was found on the background of alleles 11a (seen 10 times: three whites of European descent and seven mixed-race individuals) and 12a (seen once in a mixed-race individual). Because this is a rare indel (allele frequency <1%; DNA sequence data on file at AstraZeneca LP) that did not directly affect the poly-C allele as defined in this paper, no further analysis was carried out.

### Association of 3΄UTR poly-C repeat polymorphism with clinical response

The various poly-C genotype groups had similar pulmonary function measurements and asthma symptoms at baseline and during the run-in period (Table 2). The effect of the poly-C genotype on clinical response was explored. The results are presented in order of increasing total repeat length (the sum of an individual’s two alleles).

The proportion of patients in each poly-C genotype group who experienced a severe asthma exacerbation was between 5% and 19% (Table 3). There was variability in the incidence and rate of exacerbations across the genotype groups. Both the proportion of patients experiencing a severe asthma exacerbation and the time to first exacerbation were not statistically significant across poly-C genotype groups. Due to the overall sample sizes and the influence of a small number of patients with multiple exacerbations in some of the genotype groups, there was no clear evidence that the incidence or rate of exacerbations occurred more frequently in any particular genotype group, or with one or two copies of any particular allele.

**Table 3:** Number of patients with severe asthma exacerbations in each poly-C repeat genotype group

**Poly-C genotype**	10a/ 11a	10a/ 12a	11a/ 11a	10a/ 13b	11a/ 12a	11a/ 13b	12a/ 12a	11a/ 14b	12a/ 13b	12a/ 14b	13b/ 13b	13b/ 14b	Genotypes <1%	Undetermined
Patients, n	44	57	225	62	314	418	136	74	356	75	211	119	98	58
Patients with ≥1 severe exacerbation, n (%)	4 (9)	3 (5)	26 (12)	5 (8)	29 (9)	48 (11)	12 (9)	10 (14)	32 (9)	14 (19)	21 (10)	19 (16)	13 (13)	7 (12)
Total number of severe exacerbations	4	3	50	8	43	66	14	12	39	25	25	22	16	21
Number of exacerbations per patient/6 months	0.13	0.06	0.32	0.14	0.15	0.24	0.11	0.18	0.13	0.37	0.16	0.20	0.18	0.39

Severe exacerbations were defined as deterioration in asthma leading to hospitalization, emergency department treatment, or oral glucocorticosteroid treatment lasting three or more days. Data includes all patients with available data of all races and all treatment arms combined (total n = 2,247; all patients with data postrandomization).

**Figure 2:**
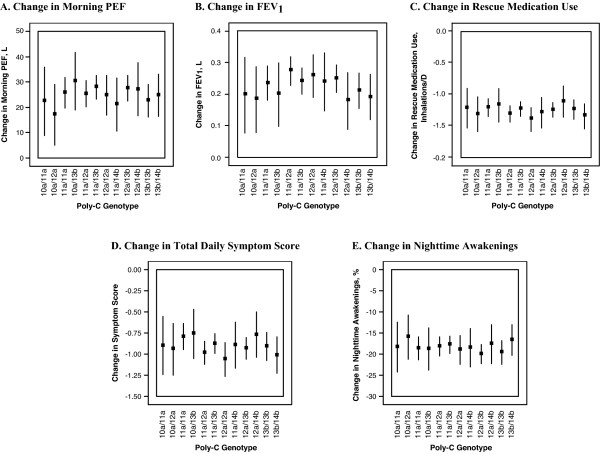
**Change in morning PEF, FEV**_**1,**_** rescue medication use, symptom scores, and nighttime awakenings by poly-C repeat genotype.** Data includes patients with poly-C repeat genotype frequencies >1% for all races and treatments with available data. Plots show least square means and 95% confidence intervals for each poly-C repeat genotype group from ANOVA models. Models include baseline, treatment, and country as covariates. For change in morning PEF (**A**), FEV_1_ (**B**), rescue medication use (**C**), daily symptom score (**D**), and nighttime awakenings (E), the total number of patients was 2,094, with 10a/11a n = 44, 10a/12a n = 57, 10a/13b n = 62, 11a/11a n = 225, 11a/12a n = 314, 11a/13b n = 418, 11a/14b n = 74, 12a/12a n = 136, 12a/13b n = 357, 12a/14b n = 77, 13b/13b n = 211, and 13b/14b n = 119. For FEV_1_ (B), the total number of patients was 2,041, with 10a/11a n = 41, 10a/12a n = 56, 10a/13b n = 60, 11a/11a n = 219, 11a/12a n = 305, 11a/13b n = 406, 11a/14b n = 73, 12a/12a n = 136, 12a/13b n = 349, 12a/14b n = 75, 13b/13b n = 202, and 13b/14b n = 110. ANOVA = analysis of variance; FEV_1_ = forced expiratory volume in one second; PEF = peak expiratory flow.

Patients in all poly-C genotype groups showed an improvement in both morning PEF and FEV_1_ after treatment (Figure 2). There is no evidence that poly-C repeat genotype affects the change from baseline to the average during the treatment period in morning PEF and FEV_1_. Similarly, patients in all poly-C genotype groups experienced a decrease in asthma symptom score, rescue medication use, and nighttime awakenings due to asthma after treatment, and there is no evidence that poly-C repeat genotype affects changes in asthma symptoms with therapy. In addition, there was no indication of any trend between genotypes of increasing allele length and changes in pulmonary function response or asthma symptoms (Figure 2; genotypes shown in order of increasing total allele length). Inclusion of the *ADRB2* Gly16Arg as a covariate was not significant and did not influence the effect of the poly-C repeat on pulmonary function outcomes (whites of European descent, morning PEF: p = 0.998, FEV_1_: p = 0.7399; Asians, morning PEF: p = 0.4933, FEV_1_: p = 0.3198).

Due to the small number of serious adverse events, discontinuations due to adverse events [13], and the complexity of the poly-C repeat region variation, it was not meaningful to explore the relationship between poly-C genotype and safety outcomes.

## Discussion

The purpose of this study was to characterize the sequence diversity in the *ADRB2* poly-C repeat region, because this diversity may have an impact on response to ICS/LABA combination therapy through changes in the regulation and expression of the β_2_-adrenergic receptor. The results of this study did not show an effect of *ADRB2* 3′UTR poly-C repeat polymorphism on the effectiveness of LABA given in combination with ICS to patients with asthma. There was no indication that a particular poly-C genotype was associated with a therapeutic outcome different from other genotypes. All genotypes had comparable improvements in pulmonary function and frequency of exacerbations at the end of the treatment period.

The 3΄UTR of *ADRB2* contains regions that may regulate gene expression and translation [20-22]. Variation in this poly-C region may influence gene regulation because of its proximity to mRNA regulatory motifs, including an AU-rich element [16]. Micro-RNA−mediated silencing of gene expression involves the base pairing of micro-RNAs with the 3′UTRs of their target mRNAs [23]. Therefore, it is possible that variation in the length of the poly-C repeat and interruptions in the poly-C repeat may have functional consequences if this 3′UTR region is a recognition site for poly-C−binding proteins that have a role in mRNA stabilization, translational activation, and translational silencing [24].

The poly-C sequence context of the triallelic SNP at +1275 (rs1042721) and other SNPs within the poly-C region (Figure 1) cannot be determined with the fragment-length−based genotyping method used by Hawkins and coworkers [16]. In this study, a novel sequencing-based genotyping method was developed to accurately genotype the alleles present in the poly-C repeat region of *ADRB2*. The method involved the use of a mismatched PCR primer, where a ‘C’ nucleotide was changed to a ‘T’ nucleotide to interrupt the poly-C repeat and therefore disrupt the secondary structure of the product. The ‘T’ nucleotide also acts as a ‘marker’ to help determine the length of the repeat from sequence data and aids genotyping of heterozygous sequences where the differing poly-C repeat lengths cause frame shifts in the DNA sequence data. Further characterization of the sequence diversity in the *ADRB2* 3΄UTR was achieved by sequencing to identify SNPs within, and in close proximity to, the poly-C repeat, including substitutions that interrupt the poly-C tract. Four novel SNPs were identified (at positions +1269, +1271, +1272, and +1276) in addition to variation in the overall poly-C repeat length from 9 to 16 nucleotides. This extends the known diversity in this region beyond that previously reported.

The poly-C repeat was characterized in 2,192 patients in this study. A total of 13 different alleles were identified and confirmed by cloning. The prevalence of the poly-C alleles differed between the population groups in this study. Resequencing identified previously uncharacterized substitution polymorphisms within the poly-C repeat region. Of the six common alleles (>5%) in this region, alleles 10a, 11a, and 12a (10-, 11- and 12-mers, respectively) each have a ‘C’ at position +1275. Alleles 13b and 14b (13- and 14-mers, respectively) each have a ‘G’ at position +1275, whilst allele 13C has an ‘A’ at position +1275. Therefore, either the length of the poly-C repeat, the substitution polymorphisms, or both, could represent the functionally important variation.

An in vitro approach showed an effect of the poly-C region on mRNA stability and steady-state cell surface expression of β_2_-adrenergic receptor [17]. These observations used constructs with uninterrupted tracts of the ‘C’ nucleotide. Resequencing data in the present study found that the longer poly-C repeat alleles (13b, 14b) were commonly interrupted by the presence of a ‘G’ nucleotide at +1275. The in vitro study only investigated the functional effect of poly-C repeat alleles of 11, 12, and 13 Cs in length [17]. Our data show that alleles with 10 to 14 nucleotides are among the most common alleles across whites of European descent and Asian populations.

Hawkins et al. reported no association between *ADRB2* haplotypes covering the whole *ADRB2* gene and pulmonary function in white patients with asthma [16]. However, a significant haplotype association was observed for African American patients with asthma compared with controls for FEV_1_/forced vital capacity (FVC) and percentage-predicted FEV_1_[16]. Haplotypes 2D and 4D (both 13-mers at the poly-C repeat) were significantly associated with FEV_1_/FVC and percentage-predicted FEV_1_[16]. Haplotypes 4C and 4D (12-mer and 13-mer at the poly-C repeat, respectively) were significantly associated with percentage-predicted FVC but with opposite Hap scores [16]. Since haplotypes 4C and 4D differ only in the length of the poly-C repeat region, the authors suggested that different lengths of the poly-C region might influence pulmonary function in African Americans [16].

In this study, there was no evidence for any clear difference in baseline pulmonary function measurements in any particular genotype group in the populations studied overall (Table 2), nor within the whites of European descent or Asian populations (data not presented). In this study, the size of the black population (n = 21) did not permit sub-group analysis in the black patients, so our conclusions cannot be compared directly to Hawkins’ observations.

In the present study, comprehensive analysis of the *ADRB2* poly-C repeat has revealed novel uncharacterized sequence variation. Association between the poly-C repeat genotypes and the clinical difference in response to ICS/LABA treatment was evaluated. The poly-C genotype groups had similar changes in pulmonary function after treatment, and there was no evidence of an association between poly-C genotype and incidence of severe asthma exacerbations. All poly-C genotype groups showed improvement in pulmonary function measurements after treatment with a combination ICS/LABA. A previous analysis of *ADRB2* haplotypes inclusive of the promoter, coding, and 3′UTR regions showed differences in *ADRB2* expression and ligand-dependent β_2_-adrenergic receptor downregulation between these haplotypes [25]. The authors suggested that patients with certain *ADRB2* haplotypes may exhibit phenotypes that differ with regard to initial treatment response or risk of tachyphylaxis [25]. An earlier analysis of 11 polymorphisms from within the *ADRB2* coding region and up to 5 kb in the 5′UTR region by Bleecker et al. included 3 of the haplotypes examined by Panebra et al. [25] and showed no differences between these haplotypes in the clinical response to ICS/LABA treatment based on measures of pulmonary function and asthma exacerbations in studies of up to 7 months in duration [13].

## Conclusions

This study describes a novel sequencing-based genotyping approach to determine the alleles present in a poly-C–rich region of the *ADRB2* 3′UTR. Results show major variation in this region of the gene; however, the variation in the 3′UTR of *ADRB2* did not affect baseline pulmonary function or therapeutic responses to ICS/LABA combination therapy in this study. Thus, *ADRB2* 3′UTR genotype does not predict therapeutic responses to ICS/LABA combination therapy.

## Abbreviations

ADRB2: β2-adrenergic receptor gene; ANOVA: Analysis of variance; DPI: Dry powder inhaler; FEV1: Forced expiratory volume in one second; FVC: Forced vital capacity; ICS: Inhaled corticosteroid; LABA: Long-acting β2-adrenergic agonist; PCR: Polymerase chain reaction; PEF: Peak expiratory flow; SABA: Short-acting β2-adrenergic agonist; SNP: Single-nucleotide polymorphism; UTR: Untranslated region.

## Misc

Study supported by AstraZeneca LP Wilmington, Delaware, USA, and AstraZeneca, Alderley Park, Cheshire, UK. Drs. Bleecker and Meyers were supported by NIH grant HL 056899.

## Competing interests

Helen Ambrose, Rachael Lawrance, Carl Cresswell, and Mitchell Goldman are AstraZeneca employees and stockholders. Eugene R Bleecker has received grant support to perform clinical trials from AstraZeneca and GlaxoSmithKline administered by his employer, Wake Forest Health Sciences, for both companies; has served as a consultant with AstraZeneca, MedImmune, and GlaxoSmithKline; and has presented CME and other lectures sponsored by AstraZeneca and GlaxoSmithKline. Deborah A Meyers has received grant funding from AstraZeneca for directing a 2-day postgraduate course on human genetics and pharmacogenetics in 2006 and 2007 and is a consultant with MedImmune.

## Authors’ contributions

ERB and DAM contributed substantially to the interpretation of the data and to drafting the manuscript and revising it critically for important intellectual content, and they provided final approval for the version to be published. MG and HJA contributed substantially to the conception and design of the study, interpretation of the data, and revising the manuscript critically for important intellectual content; they also provided final approval for the version to be published. RML contributed substantially to the development of the statistical analysis plan and to analysis and interpretation of the data; she also provided final approval for the version to be published. CJC identified a novel solution for DNA sequencing, contributed substantially to the generation of genotype data and to drafting the manuscript, and provided final approval for the version to be published. All authors read and approved the final manuscript.

## Financial disclosures

Rachael Lawrance, Carl Cresswell, Mitchell Goldman, and Helen Ambrose are AstraZeneca employees and stockholders.

Study supported by AstraZeneca LP Wilmington, Delaware, USA, and AstraZeneca, Alderley Park, Cheshire, UK. Drs. Bleecker and Meyers were supported by NIH grant HL 056899.

## Supplementary Material

Additional file 1 Online Repository [26-31] Click here for file
