# Supplementary material for: Effect of β2-adrenergic receptor gene (ADRB2) 3′ untranslated region polymorphisms on inhaled corticosteroid/long-acting β2-adrenergic agonist response
Source: Respir Res. 2012 May 4;13(1):37. doi: 10.1186/1465-9921-13-37 (PMC3441247; doi:10.1186/1465-9921-13-37)
Supplement: Additional file 1 — Online Repository [26-31] [file 1465-9921-13-37-S1.doc]

**Online Repository**

**Effect of β_2_-adrenergic receptor gene** (***ADRB2)* 3´-untranslated region polymorphisms on inhaled corticosteroid/long-acting β_2_-adrenergic agonist response**

Helen J. Ambrose^1^, Rachael M. Lawrance^1^, Carl J. Cresswell^1^, Mitchell Goldman^2^, Deborah A. Meyers^3^, and Eugene R. Bleecker^3^

^1^AstraZeneca, Alderley Park, UK

^2^AstraZeneca LP, Wilmington, Delaware, USA

^3^Wake Forest University Health Sciences, Winston-Salem, North Carolina, USA

**Introduction**

Studies have not assessed the effect of diversity in the poly-C repeat on the 3´ untranslated region (3´UTR) of the β_2_-adrenergic receptor gene (*ADRB2*). Repeats in DNA sequence of a single nucleotide, known as mononucleotide repeats (MNRs), commonly are found throughout the human genome [1,2]. Moreover, simple sequence repeats (SSRs), such as di-, tri- and tetra-nucleotide repeats, are used frequently as markers in human genetic studies [1,2]. These sequence repeats typically are genotyped based on the sizing of radioactive- or fluorescent-labeled primer extension products [2]. However, single-nucleotide polymorphisms (SNPs) have been identified within MNR regions in studies of diversity in single genes [3,4]. In addition to the variation in the length of the poly-C repeat in the *ADRB2* 3´UTR, public SNP databases indicate the presence of additional nucleotide substitutions in this region [5]. None of the fragment length–based methods published to date for genotype determination of MNR regions allow the detection or characterization of substitution polymorphisms within the repeat region.

Although sequencing is considered to be the gold standard in polymorphism detection, it is limited in the analysis of long mononucleotide repeats. Sanchez-Cespedes et al. used sequencing to analyze the mitochondrial D310 polymorphism after amplification and cloning. The D310 region consists of two poly-C stretches interrupted by a thymidine nucleotide, a crucial factor that enabled the successful analysis of this simple repeat [6].

**Methods**

DNA sequencing

The *ADRB2* 3´UTR region was refractory to genotyping using standard polymerase chain reaction (PCR) sequencing protocols, despite the addition of DMSO and other approaches to optimize sequencing of GC-rich regions. The contributory factor may have been the presence of the poly-C repeat, resulting in secondary structure formation. To sequence through the poly-C repeat (rs34522894 and rs45580732), a mismatched 5΄ primer was used to introduce a ‘T’ nucleotide 3 bp from the end of the primer, at position +1269, relative to the ATG start codon of the *ADRB2* mRNA reference sequence (Genbank Accession M15169.1) in the first round of PCR, to disrupt the secondary structure (Figure 1 in main text). 295bp PCR products were amplified with forward mismatched PCR primer; 5΄ GCAGTTTTTCTACTTTTAAAGACCCTCC 3΄, and reverse PCR primer; 5´ACTGTAAAACGACGGCCAGTGCAGACTCAGGTCCTCTAGGAC 3´, using ReddyMix^TM^ containing Thermoprime Plus DNA polymerase (Abgene, UK) and touch-down PCR reaction conditions. Mismatch primers are central to many PCR-based techniques for the detection and genotyping of polymorphisms, as exemplified by the amplification-refractory mutation system (ARMS) [7]. The reverse PCR primer included a M13 ‘tag’ (underlined) that was used to sequence each PCR product. DNA sequence data was obtained using BigDye Terminator v3.1 chemistry on an ABI3730xl DNA Analyzer (Applied Biosystems, Foster City, California, USA). Due to the complexity of the sequencing data, two operators independently determined the genotype data.

Because there is evidence from SNP databases of nucleotide substitutions in the poly-C region, it was important to eliminate the effect of rare proximal SNPs within the mismatched PCR primer that may result in hemizygous sequence data due to PCR amplification failure in alleles where additional polymorphisms are present. Thus, a standard sequencing reaction was performed to characterize other polymorphisms within the mismatched primer region and to screen for additional polymorphisms in the *ADRB2* 3´UTR. A 480bp PCR product was generated (Figure 1 in main text) using Forward PCR primer 5´-ACTGTAAAACGACGGCCAGTCTACTCCAGCAACGGCAACACAGG -3´ and Reverse PCR primer 5´-GCAGACTCAGGTCCTCTAGGAC -3´ and sequenced using the M13 ‘tag’ (underlined). Patients with nucleotide substitutions (SNPs) within the 5´ mismatched primer region were not assigned poly-C genotypes for analysis (n = 27).

The sequencing of cloned PCR products was used to validate the observed poly-C repeat sequence diversity. Independent PCR products, generated from a selection of patients representative of each of the different poly-C alleles and genotypes, were cloned. Cloning was carried out using the Invitrogen pVP22 TOPO^TM^ TA Expression kit according to the manufacturer guidelines (Invitrogen Corporation, Carlsbad, California, USA). Cloned PCR products, sequenced using primers described above, generated identical sequencing patterns to the initial sequencing results. This confirmed that the rare poly-C repeat alleles were unlikely to have been generated by PCR artifacts, such as Taq polymerase errors or sequencing errors. The sequencing of cloned PCR products also validated the novel ‘A’ insertion polymorphisms, directly 3΄ of the poly-C region, at position 1280 relative to M15169.1.

**References**

1. Katti MV, Ranjekar PK, Gupta VS: **Differential distribution of simple sequence repeats in eukaryotic genome sequences.** *Mol Biol Evol* 2001, **18:**1161-1167.
2. Cohen H, Danin-Poleg Y, Cohen CJ, Sprecher E, Darvasi A, Kashi Y: **Mono-nucleotide repeats (MNRs): a neglected polymorphism for generating high density genetic maps *in silico*.** *Hum Genet* 2004, **115:**213-220.
3. Hawkins GA, Tantisira K, Meyers DA, Ampleford EJ, Moore WC, Klanderman B, Liggett SB, Peters SP, Weiss ST, Bleecker ER: **Sequence, haplotype, and association analysis of ADRβ2 in a multiethnic asthma case-control study.** *Am J Respir Crit Care Med* 2006, **174:**1101-1109.
4. Nickerson DA, Taylor SL, Weiss KM, Clark AG, Hutchinson RG, Stengård J, Salomaa V, Vartiainen E, Boerwinkle E, Sing CF: **DNA sequence diversity in a 9.7-kb region of the human lipoprotein lipase gene.** *Nat Genet* 1998, **19:**233-240.
5. **dbSNP Database** [http://www.ncbi.nlm.nih.gov/projects/SNP/]
6. Sanchez-Cespedes M, Parrella P, Nomoto S, Cohen D, Xiao Yan, Esteller M, Jeronimo C, Jordan RC, Nicol T, Koch WM, Schoenberg M, Mazzarelli P, Fazio VM, Sidransky D: **Identification of a mononucleotide repeat as a major target for mitochondrial DNA alterations in human tumors.** *Cancer Res* 2001, **61:**7015-7019.
7. Newton CR, Graham A, Heptinstall LE, Powell SJ, Summers C, Kalsheker N, Smith JC, Markham AF: **Analysis of any point mutation in DNA. The amplification refractory mutation system (ARMS).** *Nucleic Acids Res* 1989, **17:**2503-2516.
